# Risk and course of COVID-19 in immunosuppressed patients with myasthenia gravis

**DOI:** 10.1007/s00415-022-11389-0

**Published:** 2022-09-27

**Authors:** Frauke Stascheit, Ulrike Grittner, Sarah Hoffmann, Philipp Mergenthaler, Michael Schroeter, Tobias Ruck, Mark Pawlitzki, Franz Blaes, Julia Kaiser, Ulrike Schara, Adela Della-Marina, Andrea Thieme, Tim Hagenacker, Christian Jacobi, Benjamin Berger, Peter P. Urban, Karl Christian Knop, Berthold Schalke, De-Hyung Lee, Petra Kalischewski, Heinz Wiendl, Andreas Meisel

**Affiliations:** 1 Department of Neurology with Experimental Neurology, Charité — Universitätsmedizin Berlin, Charitéplatz 1, 10117 Berlin, Germany; 2 NeuroCure Clinical Research Center, Charité — Universitätsmedizin Berlin, Berlin, Germany; 3 Center for Stroke Research Berlin, Charité — Universitätsmedizin Berlin, Berlin, Germany; 4 Institute of Biometry and Clinical Epidemiology, Charité-Universitätsmedizin Berlin, Berlin, Germany; 5 Berlin Institute of Health at Charité – Universitätsmedizin Berlin, Charitéplatz 1, 10117 Berlin, Germany; 6 Department of Neurology, University of Cologne and University Hospital, Cologne, Germany; 7 Department of Neurology, Medical Faculty, Heinrich Heine University Düsseldorf, Düsseldorf, Germany; 8 Department of Neurology, Kreiskrankenhaus Oberberg GmbH, Oberberg, Germany; 9 Department of Neurology, LVR Klinik Bonn, Bonn, Germany; 10 Department of Neuropediatric, University of Duisburg-Essen, Essen, Germany; 11 Department of Neurology, Helios Hospital Erfurt, Erfurt, Germany; 12 Department of Neurology Center for Translational Neuro- and Behavioral Sciences (C-TNBS), University Medicine Essen, Essen, Germany; 13 Department of Neurology, Sankt Katharinen Krankenhaus GmbH, Frankfurt, Germany; 14 Department of Neurology, Helios Hospital Pforzheim, Pforzheim, Germany; 15 Clinic of Neurology and Neurophysiology, Medical Center, Faculty of Medicine, University of Freiburg, Freiburg, Germany; 16 Department of Neurology, Asklepios Hospital Hamburg Barmbek, Hamburg, Germany; 17 Neurological Outpatient Department Neuer Wall, Hamburg, Germany; 18 Department of Neurology, University of Regensburg, Regensburg, Germany; 19 Neurological Outpatient Department, Leipzig, Germany; 20 Department of Neurology, University of Münster, Münster, Germany

**Keywords:** Myasthenia gravis, COVID-19, Immunosuppressive therapies, Outcome, German myasthenia gravis registry

## Abstract

**Background:**

Patients with myasthenia gravis (MG) are potentially prone for a severe COVID-19 course, but there are limited real-world data available on the risk associated with COVID-19 for patients with MG. Here, we investigate whether current immunosuppressive therapy (IST) influences the risk of SARS-CoV-2 infection and COVID-19 severity.

**Methods:**

Data from the German myasthenia gravis registry were analyzed from May 2020 until June 2021 and included patient demographics, MG disease duration, comorbidities, current IST use, COVID-19 characteristics, and outcomes. Propensity score matching was employed to match MG patients with IST to those without, and multivariable binary logistic regression models were used to determine associations between IST with (1) symptomatic SARS-CoV-2 infection and (2) severe COVID-19 course, as measured by hospitalization or death.

**Results:**

Of 1379 patients with MG, 95 (7%) patients (mean age 58 (standard deviation [SD] 18) presented with COVID-19, of which 76 (80%) received IST at time of infection. 32 patients (34%) were hospitalized due to COVID-19; a total of 11 patients (12%) died. IST was a risk factor for hospitalization or death in the group of COVID-19-affected MG patients (odds ratio [OR] 3.04, 95% confidence interval [CI] = 1.02–9.06, *p* = 0.046), but current IST was not associated with a higher risk for SARS-CoV-2 infection itself.

**Discussion:**

In this national MG cohort study, current IST use was a risk factor for a severe disease course of COVID-19 but not for SARS-CoV-2 infection itself. These data support the consequent implementation of effective strategies to prevent COVID-19 in this high-risk group.

**Trial registration information:**

German clinical trial registry (https://www.drks.de), DRKS00024099, first patient enrolled: February 4th, 2019.

## Introduction

Coronavirus disease 2019 (COVID-19) is a global pandemic and raised concerns about the risk of severe infections in patients with myasthenia gravis (MG) due to several factors, such as preexistent bulbar and respiratory muscle fatigability, exacerbation of symptoms due to infections [16, 28], and an immunocompromised state due to the immunosuppressive therapies (IST) that can be found in up to 80% of patients with MG [33]. There is increasing evidence that patients receiving corticosteroids [17, 49] or rituximab for treatment of different rheumatological [5, 43] and neurological conditions [44, 47] have a higher risk of severe disease courses of COVID-19 than patients without such medications. In addition, many comorbidities associated with a high mortality in COVID-19 [11], such as cardiovascular diseases and type II diabetes, are common among patients with MG [14].

On the other hand, it is well recognized that the severity and outcome of COVID-19 might be associated with the excessive production of pro-inflammatory cytokines [51] and IST is considered as treatment of choice in patients severely affected by COVID-19 [34]. Thus, the associated risk of COVID-19 severity related to prior IST remains uncertain.

To date, there are limited real-world data on the risk associated with COVID-19 for patients with MG [2, 6, 8, 25, 36, 46]. Management of patients with MG during COVID-19 pandemic has been guided by expert consensus [24]. Apart from a series of case reports [2, 6, 8], the largest studies to date, the international CARE-MG registry [36], and the Czech-MG study [25] reported a mortality of COVID-19 in patients with MG of 24% and 11%, respectively. These data suggest a higher mortality in MG than in the general population with COVID-19 infection, where it is approximately 2% [12, 15, 53]. However, these numbers may be affected by reporting bias, because severe courses are usually hospitalized and therefore easier to capture. This likely has a greater impact when rare diseases are analyzed in small patient populations. Therefore, it is important to understand whether IST is an additional factor favoring the risk of SARS-CoV-2 infection as well as disease severity and poor outcome of COVID-19 in patients with MG. Taking into account known risk factors for severe COVID-19 courses, such as higher age, sex, and comorbidities [15, 53], we here investigated whether patients with MG receiving IST are at higher risk for symptomatic SARS-CoV-2 infection and prone to a worse outcome in case of COVID-19 compared to patients with MG without IST.

## Methods

### Study design and data collection

This multicenter national cohort study analyzed data from the German Myasthenia gravis Registry (MyaReg), which was established in February 2019 by the national patient support organization for patients with MG (German Myasthenia gravis Society; DMG). MyaReg is assessing longitudinal clinical data on diagnostics, therapy, adverse events, socioeconomic status, and patient-reported outcome parameters of patients with myasthenic syndromes including MG and Lambert–Eaton myasthenic syndrome (LEMS), which were diagnosed based on the current German guidelines [35].

Patients were followed up with entry into the registry in specialized MG clinics on an ongoing basis until June 2021. Socio-demographics (age, sex), MG disease duration, current MG-specific medication, which includes cholinesterase inhibitors, first-line IST (corticosteroids, azathioprine, mycophenolate mofetil, methotrexate, cyclosporine), escalation therapy (rituximab, eculizumab), history of thymectomy, and comorbidities were collected in an electronic database (asthesis^®^). SARS-CoV-2 infection-related data encompassed COVID-19 severity (hospitalization, stay on intensive care unit, invasive/non-invasive ventilation, myasthenic exacerbation/crisis, exacerbation therapies, change of preexistent IST, antiviral therapy), and outcome (death, rehabilitation, nursing home, discharge to home). Exclusion criteria were missing information on COVID-19 diagnosis (by polymerase chain reaction or antibody testing), MG disease duration, comorbidities, and current MG-specific medication.

### Outcome

Endpoints were symptomatic SARS-CoV-2 infection (COVID-19 of any severity) and severity of COVID-19. The severity of COVID-19 course was classified as mild (defined as outpatient treatment), moderate (defined as hospitalization without ICU treatment), and severe (defined as ICU treatment and deceased). We analyzed whether a current IST use was associated with (1) a symptomatic SARS-CoV-2 infection, and (2) with a worse course of COVID-19 defined by hospitalization or death.

### Statistical analysis

As descriptive statistics, means and standard deviation (SD) or median and interquartile ranges (IQR) for continuous variables, and absolute and relative frequencies for nominal data were reported. Standardized mean differences (SMD) were calculated as standardized effect sizes. To evaluate the association of IST with the risk of SARS-CoV-2 infection, propensity score matching was employed to match MG patients with IST (exposure) to those without IST. Variables that were used for matching were age, sex, specific diagnosis of myasthenic syndrome, arterial hypertension, thymectomy, heart failure, obesity, chronic obstructive pulmonary disease (COPD), diabetes, and Myasthenia Gravis Foundation of America (MGFA) score. A caliper of 0.2 (of the standard deviation of the logit) was applied [4]. The matching ratio for matching patients with and without IST ranged from 1 to 9, resulting in 203 patients without IST matched to 949 patients with IST. Within the matched subgroup of patients with MG, a multivariable binary logistic regression and a generalized estimation equation (GEE) regression model with adjustment for age, sex, presence of comorbidities, and MGFA clinical classification score was used to analyze the association of IST to risk of infection. Additionally, to evaluate the association of current IST use and COVID-19 severity within the group of COVID-19 patients, SMD were calculated, and multivariable binary logistic regression models adjusted for age, sex and the presence of comorbidities were performed. Patients receiving eculizumab were excluded for risk analysis due to mode of action and the small number of subjects treated with eculizumab in our population. Odds ratios (OR) and 95% confidence interval (CI) are reported. Statistical analyses were performed using SPSS (IBM Corp. Released 2020. IBM SPSS Statistics for Windows, Version 27.0. Armonk, NY: IBM Corp) and R (R Core Team 2020) [50], package MatchIt [18], and the R package geepack [19].

### Data availability

All analyzed data are presented in the manuscript and are available on reasonable request from qualified investigators.

## Results

### Demographics and clinical characteristics

We included 1379 patients with myasthenic syndromes (mean age 59 [SD 18]). Because of missing details on COVID-19 diagnosis, comorbidities, and current MG-specific therapy status, four patients with COVID-19 had to be excluded. Overall, 95 patients with MG and COVID-19 could be included for further analysis, of which the majority was infected with SARS-CoV-2 until March 2021, and only 2% (*n* = 2) were partly vaccinated against SARS-CoV-2 at the time of infection. MG patients with COVID-19 were comparable to those without COVID-19 regarding age (58 vs. 59 years, SMD: 0.04), sex (41% males vs. 44% males, SMD: 0.05) and disease duration (median 65 vs. 55 months, SMD: 0.13). Atrial fibrillation (11% vs. 5%, SMD: 0.20) and COPD (11% vs. 4%, SMD: 0.24) were more prevalent in MG patients with SARS-CoV-2 infection compared to those without infection. The vast majority of MG patients with and without COVID-19 received IST (80% vs 84%, SMD: 0.19), of which first-line IST (azathioprine, mycophenolate mofetil, methotrexate, cyclosporine) was the most common (52% vs. 61%), followed by corticosteroid monotherapy (19% vs. 16%) and escalation therapy with rituximab (7.4 vs. 6.7) or eculizumab (2.1% vs. 1.4%). A history of thymectomy was similar in both patient groups (43% vs 40%).

### Risk of SARS-CoV-2 infection is not associated with immunosuppressive treatment

To investigate whether a current use of corticosteroid monotherapy, first line, and escalation (only rituximab) IST was associated with SARS-CoV-2 infection, we compared MG patients with and without IST including corticosteroids considering relevant covariates (Table 1). In the group of all MG patients before matching, SARS-CoV-2 infections occurred in 76 of 1167 patients treated with IST (6.5%) compared to 19 of 221 treated without IST (8.6%; Table 1). In the matched subgroup, SARS-CoV-2 infections occurred in 5.1% of patients treated with IST (*n* = 949 in 203 clusters) compared to 7.9% of those treated without IST (*n* = 203). A multivariable binary logistic regression model in the non-matched MG group as well as a GEE model in the matched MG group revealed no substantial association of current IST to the risk of symptomatic SARS-CoV-2 infection (all OR below 1, all 95% CIs included 1; Table 2).

**Table 1:** Characteristics of patients with myasthenia gravis and with or without immunosuppressive therapy before and after propensity score matching

	Patients with IST	Patients without IST	SMD	Matched patients with IST^a^	Matched patients without IST	SMD
*n*	1164	215		949 patients in 203 clusters	203	
Sex male, *n* (%)	531 (45.5%)	71 (32.1%)	0.28	66.6 (32.8%)	68 (33.5%)	0.02
Age in years, mean (SD)	59 (18)	56 (19)	0.19	57 (10)	57 (18)	0.02
diagnosis						
MG	1145 (98.4%)	208 (96.7%)	0.23	196.3 (96.7%)	197 (97.0%)	
LEMS	19 (1.6%)	7 (3.3%)		5.9 (2.9%)	5 (2.5%)	
Disease duration in months, median (IQR) (34 missing’s)	59 (23–123)	42 (10–133)	0.05	79 (55–132)	42 (11–134)	0.02
Thymectomy, n (%) (4 missing’s)	473 (40.7%)	79 (35.7%)	0.10	82.0 (40.4%)	78 (38.4%)	0.05
MGFA (13 missing’s)			0.53			0.05
0	61 (5.0%)	12 (5.5%)		11.8 (5.8%)	11 (5.4%)	
I	210 (18.2%)	73 (33.3%)		62.7 (30.9%)	67 (33.0%)	
II A	298 (25.8%)	57 (26.0%)		58.3 (28.7%)	55 (27.1%)	
II B	310 (26.8%)	60 (27.4%)		58.3 (28.7%)	57 (28.1%)	
III A	92 (8.0%)	7 (3.2%)		3.2 (1.6%)	4 (2.0%)	
III B	121 (10.5%)	7 (3.2%)		7.1 (3.5%)	7 (3.4%)	
IV A/IV B /V	64 (5.5%)	3 (1.4%)		1.6 (0.8%)	2 (1.0%)	
Comorbidities present, *n* (%) (2 missing’s)	945 (81.0%)	181(81.9%)	0.02	162.0 (79.8%)	166 (81.8%)	0.06
Autoimmune disease, *n* (%)	273 (23.4%)	50 (22.6%)	0.02	52.8 (26.0%)	45 (22.2%)	0.11
Diabetes mellitus type II, *n* (%)	154 (13.2%)	28 (12.7%)	0.02	24.6 (12.1%)	26 (12.8%)	0.03
Arterial hypertension, *n* (%)	413 (35.4%)	73 (33.0%)	0.05	65.4 (32.2%)	71 (35.0%)	0.07
Heart failure, *n* (%)	23 (2.0%)	1 (0.5%)	0.14	1.2 (0.6%)	1 (0.5%)	0.02
Atrial fibrillation, *n* (%)	67 (5.7%)	10 (4.5%)	0.06	9.3 (4.6%)	10 (4.9%)	0.02
Obesity, *n* (%)	24 (2.1%)	5 (2.3%)	0.01	3.9 (1.9%)	4 (2.0%)	0.01
COPD, *n* (%)	62 (5.3%)	3 (1.4%)	0.22	4.1 (2.0%)	3 (1.5%)	0.05
Asthma, *n* (%)	119 (10.2%)	28 (12.7%)	0.08	23.3 (11.5%)	27 (13.3%)	0.07
Cancer, *n* (%)	123 (10.5%)	22 (10.0%)	0.02	19.7 (9.7%)	22 (10.8%)	0.05
Stroke, *n* (%)	34 (2.9%)	7 (3.2%)	0.01	5.9 (2.9%)	7 (3.4%)	0.04
Dementia, *n* (%)	6 (0.5%)	1 (0.5%)	< 0.01	0.8 (0.4%)	1 (0.5%)	0.02
SARS-CoV-2 infection	76 (6.5%)	19 (8.6%)	0.08	10.4 (5.1%)	16 (7.9%)	0.13

Data are presented as mean (SD), *n* (%) or median (IQR) showing differences in numbers and characteristics of MG patients with or without immunosuppressive therapy (IST) before and after propensity score matching and were compared by standardized mean differences (SMD). Variables used for matching were age, diagnosis, thymectomy, presence of autoimmune disease, diabetes, arterial hypertension, heart failure, atrial fibrillation, obesity, chronic obstructive lung disease (COPD), asthma, cancer, stroke, and dementia. For COPD and atrial fibrillation exact matching was used. For age, a caliper of 0.5 was chosen. Disease duration is the time from diagnosis until examination date

Abbreviations: *COPD* chronic obstructive pulmonary disease, *LEMS* Lambert–Eaton-Myasthenic Syndrome, *IST*  immunosuppressive therapy, *IQR* interquartile range, *MG* myasthenia gravis, *MGFA *Myasthenia gravis foundation of America classification, *n* number of included patients, *SD* standard deviation, *SMD* standardized mean differences

^a^Based on average measures on cluster level

^b^Based on log-transformed values

**Table 2:** Multivariable binary logistic regression model in total and generalized estimation equation binary logistic regression model in matched myasthenia gravis patients for SARS-CoV-2 infection with regard to immunosuppression status

	Non-matched MG patients (*n* = 1352^a^) Nagelkerke *R*^2^ = 0.02		Matched MG patients (*n* = 203 groups, 936 individuals^a^) Marginal *R*^2^ = 0.01	
	OR (95% CI) for COVID-19 infection	*p*	OR (95% CI) for COVID-19 infection	*p*
No corticosteroids and no immune suppression (reference)	1		1	
Only corticosteroids	0.82 (0.41–1.66)	0.589	0.74 (0.35–1.60)	0.445
First line IST (AZT, MMF, MTX, CSA)	0.56 (0.31–1.01)	0.052	0.60 (0.32–1.12)	0.107
Escalation therapy with rituximab	0.59 (0.23–1.56)	0.289	0.85 (0.29–2.51)	0.769
Age in decades	1.00 (0.99–1.02)	0.692	–	
Sex male, ref: female	1.10 (0.68–2.09)	0.541	–	
Comorbidities present, ref: no comorbidities present	1.19 (0.68–2.09)	0.541	–	
MGFA, ref: 0			–	
I	0.39 (0.16–0.97)	0.043	–	
II A	0.53 (0.22–1.25)	0.146	–	
II B	0.50 (0.21–1.18)	0.112	–	
III A	0.80 (0.28–2.28)	0.672	–	
III B	0.70 (0.25–1.94)	0.491	–	
IV A/IV B/V	1.10 (0.36–3.33)	0.868	–	

Odds ratios (OR) and 95% confidence interval (CI) for SARS-CoV-2-infected myasthenia gravis (MG) patients from multivariable binary logistic regression (left side) adjusted for age, sex, MGFA score, and thymectomy status (*n* = 1352, Nagelkerke R Square: 0.02), and from generalized estimation equation (GEE) binary logistic regression model (right side) after propensity score matching (*n* = 203 matched groups, 936 individuals) without further adjustment

Abbreviations: *AZT* azathioprine, *CI* confidence interval, *CSA* cyclosporine A, *GEE* generalized estimation equation, *IST* immunosuppressive therapy, *MG* myasthenia gravis, *MGFA* Myasthenia gravis foundation of America classification, *MMF* mycophenolate mofetil, *MTX* methotrexate, *n* number of included patients, *OR* odds ratio

^a^Patients treated with eculizumab (*n* = 20) or missing covariate data (*n* = 16) were excluded from matched and non-matched analysis

### Immunosuppressive treatment is associated with COVID-19 severity

To investigate a potential impact of current IST on the course of COVID-19, we included only the subset of all COVID-19-affected MG patients (*n* = 95) in the further analysis. Thirty-two (34%) of those MG patients were hospitalized. Twelve (13%) patients were admitted to the ICU, and six of them survived (Table 3). Eight patients required non-invasive ventilation or respiratory support, and ten received invasive ventilation. Myasthenic exacerbation was observed in nine patients, of which five were treated with intravenous immunoglobulins (IVIG), three with plasmapheresis, and one with high-dose corticosteroids (data not shown). Eleven (12%) patients died (Table 3).

**Table 3:** Characteristics of myasthenia gravis patients with COVID-19

	Non-hospitalized patients	Hospitalized and/or deceased patients	SMD	Hospitalized patients (survivors, no intensive care)	Patients with intensive care (survivors)	Deceased patients
*n*	63	32		15	6	11
Sex male, *n* (%)	21 (33.3%)	18 (56.3%)	0.47	8 (53.3%)	4 (66.7%)	6 (54.5%)
Age in years, mean (SD)	53 (18)	68 (15)	0.94	63 (13)	66 (21)	76 (11)
Disease duration in months, median (IQR) (3 missing’s)	64 (26–123)	65 (29–163)	0.11^a^	51 (27–232)	31 (7–111)	109 (47–160)
Thymectomy, *n* (%) (4 missing’s)	28 of 60 (46.7%)	11 of 31 (35.5%)	0.23	8 (53.3%)	2 (33.3%)	1 (10.0%)
MGFA			0.83			
0	4 (6.3%)	4 (12.5%)		2 (13.3%)	1 (16.7%)	1 (9.1%)
I	12 (19.0%)	3 (9.4%)		1 (6.7%)	–	2 (18.2%)
II A	15 (23.8%)	8 (25.0%)		6 (40.0%)	–	2 (18.2%)
II B	17 (27.0%)	5 (15.6%)		3 (20.0%)	2 (33.3%)	–
III A	8 (12.7%)	1 (3.1%)		–	–	1 (9.1%)
III B	5 (7.9%)	4 (12.5%)		1 (6.7%)	3 (50.0%)	–
IV A/IV B/V	2 (3.2%)	7 (21.9%)		2 (13.3%)	–	5 (45.5%)
Comorbidities present, *n* (%)	46 (73.0%)	28 (87.5%)	0.37	12 (80.0%)	5 (83.3%)	11 (100.0%)
Autoimmune disease, *n* (%)	14 (22.2%)	5 (15.6%)	0.17	2 (13.3%)	1 (16.7%)	2 (18.2%)
Diabetes type II, *n* (%)	5 (7.9%)	5 (15.6%)	0.24	–	1 (16.7%)	4 (36.4%)
Arterial hypertension, *n* (%)	21 (33.3%)	16 (50.0%)	0.34	6 (40.0%)	4 (66.7%)	6 (54.5%)
Heart failure, *n* (%)	–	3 (9.4%)	0.46	2 (13.3%)	–	1 (9.1%)
Atrial fibrillation, *n* (%)	6 (9.5%)	4 (12.5%)	0.10	–	–	4 (36.4%)
Obesity, *n* (%)	1 (1.6%)	3 (9.4%)	0.35	1 (6.7%)	1 (16.7%)	1 (9.1%)
COPD, *n* (%)	2 (3.2%)	8 (25.0%)	0.66	3 (20.0%)	2 (33.3%)	3 (27.3%)
Asthma, *n* (%)	9 (14.3%)	2 (6.3%)	0.27	1 (6.7%)	–	1 (9.1%)
Cancer, *n* (%)	4 (6.3%)	6 (18.8%)	0.38	4 (26.7%)	–	2 (18.2%)
Stroke, *n* (%)	1 (1.6%)	2 (6.3%)	0.24	1 (6.7%)	1 (16.7%)	–
Dementia, *n* (%)	–	2 (6.3%)	0.37	–	1 (16.7%)	1 (9.1%)
Current MG-specific IST			0.48			
No immune suppression, *n* (%)	14 (22.2%)	5 (15.6%)		2 (13.3%)	1 (16.7%)	2 (18.2%)
Only steroids, *n* (%)	15 (23.8%)	3 (9.4%)		–	1 (16.7%)	2 (18.2%)
Standard IST (AZT, MMF, MTX, CSA), *n* (%)	29 (46.0%)	20 (62.5%)		11 (73.3%)	4 (66.7%)	5 (45.5%)
Escalation therapy						
Rituximab, *n* (%)	4 (6.3%)	3 (9.4%)		1 (6.7%)	–	2 (18.2%)
Eculizumab, *n* (%)	1 (1.6%)	1 (3.1%)		1 (6.7%)	–	–

Data are presented as mean (SD), n (%) or median (IQR) showing standardized mean differences (SDM) of hospitalized versus non-hospitalized MG patients with COVID-19 and clinical characteristics of hospitalized and deceased patients

Abbreviations: *AZT* azathioprine, *COPD* chronic obstructive pulmonary disease, *CSA* cyclosporine A, *IST* immunosuppressive therapy, *IQR* interquartile range, *MG* myasthenia gravis, *MGFA* Myasthenia Gravis Foundation of America classification, *MMF* mycophenolate mofetil, *MTX* methotrexate, *n* number of included patients, *SD* standard deviation, *SDM* standardized mean differences

^a^ based on log-transformed data

Patients hospitalized or deceased compared with non-hospitalized patients were older (68 vs. 53 years, SMD: 0.94), more frequently male (56% vs. 33% males, SMD: 0.47), more severely affected by MG according to MGFA classification score (SMD: 0.83), more frequently receiving MG related IST (84.4% vs. 77.8%, SMD: 0.48) and had a higher prevalence of comorbidities (88% vs. 73%, SMD: 0.37). Several known risk factors for poor outcome after COVID-19 were more frequent in the group of hospitalized or deceased patients compared with the group of non-hospitalized patients: arterial hypertension (50% vs. 33%, SMD: 0.34), COPD (25% vs. 3%, SMD: 0.66), and cancer (19% vs 6%, SMD: 0.38). A history of thymectomy (36% vs 47%; SMD: 0.23) was less frequently present in patients who were hospitalized or deceased. Disease duration (median 65 vs. 64 months, SMD: 0.11) was similar in both groups. Patients with MG who died due to COVID-19 where more likely to be older with 76 (SD 11) vs. 53 (SD 18) years and more likely to be male (55% vs. 33%) compared to non-hospitalized MG patients with COVID-19. All deceased patients had at least one concomitant disease, with arterial hypertension (*n* = 6) and type two diabetes type II (*n* = 4) being the most common. The majority of these patients had MG-specific IST at time of infection (*n* = 9), of whom 2 received rituximab (Table 3).

After pooling rituximab-treated patients (*n* = 7) with first-line IST due to the low case number of this subgroup, multivariable binary logistic regression adjusted for age, sex, and comorbidities showed that SARS-CoV-2-infected patients with MG and current IST had a higher risk for hospitalization or death in comparison to patients without IST or only corticosteroid use (OR 3.04, 95% CI 1.02–9.06, *p* = 0.046; Table 4, Model 2). Age was an independent additional risk factor for COVID-19 severity in both models (OR 1.8, 95% CI 1.22–2.64, respectively, OR 1.76, 95% CI 1.21–2.56, *p* = 0.003).

**Table 4:** Multivariable binary logistic regression models for risk of hospitalization or death in patients with COVID-19 with regard to immunosuppressive status

	Model 1 *N* = 93, Nagelkerke *R*^2^ = 0.30			Model 2 *N* = 93, Nagelkerke *R*^2^ = 0.29	
	OR (95% CI) for hospitalization or death	*p*		OR (95% CI) for hospitalization or death	*p*
No IST or only corticosteroids (reference)	1		No IST or only corticosteroids (reference)	1	
First line IST (AZT, MMF, MTX, CSA)	2.86 (0.94–8.73)	0.064	First line IST (AZT, MMF, MTX, CSA) or escalation therapy (rituximab)	3.04 (1.02–9.06)	0.046
Escalation therapy (rituximab)	5.03 (0.72–35.01)	0.103			
Age in decades	1.80 (1.22–2.64)	0.003	Age in decades	1.76 (1.21–2.56)	0.003
Sex male, ref: female	1.43 (0.50–4.04)	0.506	Sex male, ref: female	1.43 (0.51–4.04)	0.501
Comorbidities present, ref: no comorbidities present	0.85 (0.19–3.86)	0.835	Comorbidities present, ref: no comorbidities present	0.90 (0.20–4.03)	0.889

Odds ratios (OR) and 95% confidence interval (CI) for hospitalization and death within the group of COVID-19 patients. Multivariable binary logistic regression models were calculated adjusted for age, sex and the presence of comorbidities. Patients treated with eculizumab were excluded from this analysis due to mode of action. In model 2 patients receiving rituximab were included to patients receiving first-line IST due to the low case number (*n* = 7)

Abbreviations: *AZT* azathioprine, *CI* confidence interval, *CSA* cyclosporine A, *IST* immunosuppressive therapy, *MMF* mycophenolate mofetil, *MTX* methotrexate, *OR* odds ratio

## Discussion

To our knowledge, this study is to date the largest to evaluate the risk of COVID-19 in MG patients in regard to current IST use with an appropriate control group of uninfected MG patients. We identified current IST use not to be a substantial risk factor for SARS-CoV-2 infection. However, IST, including first line and escalation therapy, was a risk factor for poor COVID-19 prognosis with a higher likelihood of hospitalization and death. Corticosteroid therapy alone was not a relevant risk factor for COVID-19 severity, which is in contrast to other studies examining the impact of IST in MG for outcome after COVID-19 [25, 36, 46]. However, our data indicate that known risk factors for severe COVID-19, such as patient age [11], have a greater impact on the severity of COVID-19 in MG patients than does IST.

Although data on COVID-19 and MG are still scarce, experts agreed early in the pandemic that caution was needed in patients with MG [16, 28, 38]. First, there is the risk of exacerbation of myasthenic symptoms from SARS-CoV-2 infection, compounded by the potentially higher risk of severe courses of COVID-19 due to the immunocompromised state from IST. Approximately 80% of patients with MG receive IST [33], which is in line with our data. Second, discontinuation of IST in patients with MG would likely lead to worsening of myasthenic symptoms. Our data suggest that IST is not per se associated with a higher risk of SARS-CoV-2 infection. This might be due to MG patients with IST being more concerned about COVID-19 than MG patients without IST leading to better compliance with hygiene recommendations in Germany during the study period (e.g., lockdowns, social distancing rules) [26, 31].

Nevertheless, expert consensus agreed that especially MG patients receiving rituximab were more prone to worse COVID-19 outcome [24], as there was evidence of higher risk for hospitalization and mortality in patients with rheumatoid arthritis (RA) [13] or multiple sclerosis (MS) [47] receiving anti-CD20 therapy, which has now been confirmed by large meta-analysis studies [40, 42]. Rituximab treatment is often required long term in MG and may then be associated with a higher risk of severe infections, as recently shown in a Swedish cohort study of MS patients compared with other IST [32]. In our population, seven COVID-19-affected patients received rituximab therapy prior to their infection, of which two patients died. While the association between rituximab therapy and severe outcome is not pronounced in our study (OR 2.35; 95% CI 0.29–19.08), the Czech-MG-COVID-19 study revealed a poor outcome after COVID-19 with three deaths in four MG patients treated with rituximab (OR 35.14; 95% CI 3.2–383.9) [25]. Additionally, they found that long-term use of corticosteroids, especially at high dosages, together with rituximab and older age was associated with severity of COVID-19 progression [25], which is also observed in other autoimmune diseases such as RA [13] or MS [47]. In CARE-MG, no information about rituximab therapy was reported; however, of the 91 hospitalized patients, 89% had received IST [36].

The role of corticosteroids as a risk factor of SARS-CoV-2 infection is uncertain, as systemically administered corticosteroids are effective in reducing COVID-19 mortality and are particularly beneficial during acute respiratory distress syndrome (ARDS) [48]. However, corticosteroids may prolong viremia [22] in early COVID-19 stages, which could explain the higher proportion of hospitalized patients with ongoing corticosteroid treatment. It is important to emphasize that we also identified IST including first-line and escalation therapies, besides patient age, as important independent risk factor for severe progression of COVID-19 in patients with MG. It its well known that IST may have an attenuating effect in the second phase of severe COVID-19 to suppress or even prevent cytokine storm [7, 34, 51]. In consequence, dexamethasone is recommended in severe COVID-19 to be administered to modulate inflammation-mediated lung injury reducing progression to respiratory failure and death [20] and is routinely applied in severe COVID-19 in Germany. Standard IST used in MG care might be not as effective in suppressing cytokine storm syndrome and, on the other hand, increase the risk for severe outcome of SARS CoV-2 infection more than other IST. For example, the interleukin-6 receptor antagonist tocilizumab [41] has been approved for treatment of severe COVID-19. The C5 complement inhibitor eculizumab also shows beneficial effects in patients with severe COVID-19 [3, 9]. Eculizumab is effective and approved in refractory generalized acetylcholine receptor antibody-positive MG [21], and therefore, it might be considered a potentially beneficial treatment option for patients with MG with severe COVID-19, which is why we have not included this patient group in our risk analyses. Although IVIG and therapeutic plasma exchange have no significant benefit on outcome of patients with COVID-19 [29, 45], due to its proven effect in myasthenic crises or exacerbation [38], IVIG and therapeutic plasma exchange [10, 23, 39] should be considered a first-line treatment choice in patients with MG exacerbation in the course of COVID-19 [24, 27].

Data on the role of thymectomy for COVID-19 disease course in MG are scarce. In a systematic review of case series and cases, 5% of patients with a history of thymectomy died, whereas 17% of patients without a history of thymectomy died [1]. Also in our cohort, the proportion of patients who were hospitalized or died was lower with thymectomy in the history than without thymectomy (36% versus 47%). However, this may be biased by age, as patients with late MG do not undergo thymectomy.

The major limitation of previously published studies on COVID-19 in patients with MG is the lack of an uninfected control group. In addition, there seems to be a reporting bias towards severe cases in previous studies, as the CARE-MG study preliminarily found a mortality rate of 24% [36], which compares with 11% in the Czech-MG study [25], 12% in a systematic review [1], and 12% in our study. These differences could be due to various standards of care in the countries from which the CARE-MG data are collected. Nevertheless, the COVID-19-related mortality rate of patients with MG is significantly higher compared to other autoimmune diseases such as RA [13] and MS [47], suggesting that not only IST but also MG-specific characteristics, e.g., exacerbation of myasthenic symptoms/myasthenic crisis due to COVID-19 might influence the outcome. In our study population, 10% of COVID-19-affected patients with MG suffered from exacerbation of myasthenic symptoms, which is comparable with the Czech-MG study [25], but less than in CARE-MG with a rate of 40% [36] and the systematic review with 19% [1]. One COVID-19 patient in our cohort died during the course of a myasthenic crisis, which is within the expected range of a 10% in-hospital mortality for myasthenic crises in Germany [37, 39].

## Limitations

Our results obtained in German patients with MG may not be representative for other countries, because SARS-CoV-2 infection and COVID-19 may have varying effects worldwide due to different health care systems. Moreover, the data presented here are based on patients enrolled in a registry and therefore do not cover all COVID-19 affected patients with MG in Germany. Patients who were not treated at a specialized MG center may have a different risk regarding the course of an SARS-CoV-2 infection. However, baseline characteristics about age, disease duration, and MG-specific treatment are similar to another large MG study in Germany [30]. Although we included the main known influencing factors, such as age, sex, disease severity, and comorbidities, in the matching, we cannot exclude the risk of residual bias in propensity score matching. Moreover, we have not included the auto-antibody status in our risk analysis, which potentially could have an impact on COVID-19 disease course due to differences in immune response [52, 54]. Additionally, we cannot exclude a reporting bias for hospitalized and severe cases for patients with MG included in our study, although we minimized the risk by our multicentric approach. Due to the small numbers of COVID-19-affected patients with MG in the IST subgroups, we cannot draw conclusions about the risk with the single IST, which especially accounts for rituximab. We were unable to include data on IST used in the past, IST dosage, and duration of ongoing IST before infection in our analysis. We also did not collect data on social or economic factors (e.g., employed or retired) that could potentially influence virus exposure. Future studies with a larger number of patients with MG and COVID-19 are strongly needed to confirm our results and, in particular, to clarify questions regarding the impact of different immunosuppressant therapies.

## Conclusions

This registry-based cohort study suggests that the current use of IST does not increase the risk of SARS-CoV-2 infection per se but, together with older age, worsens the prognosis of COVID-19-affected patients with MG. Nevertheless, corticosteroid monotherapy was not a relevant risk factor for COVID-19 severity in our study. Our data indirectly support the consequent implementation of vaccination strategies, such as early booster vaccination, especially in patients with MG treated with IST to effectively prevent COVID-19 in this high-risk group. Further studies with larger patient populations are strongly needed to better understand the risk and consequences of individual immunosuppressant subgroups for patients with MG, while the COVID-19 pandemic persists.

## Data Availability

The study was conducted in accordance to the Declaration of Helsinki and the STROBE reporting guidelines.
